# Intraspinal stimulation with a silicon-based 3D chronic microelectrode array for bladder voiding in cats

**DOI:** 10.1088/1741-2552/abca13

**Published:** 2020-12-16

**Authors:** Victor Pikov, Douglas B McCreery, Martin Han

**Affiliations:** 1 Medipace, Inc., Pasadena, CA 91101, United States of America; 2 Neural Engineering Program, Huntington Medical Research Institutes, Pasadena, CA 91101, United States of America; 3 Biomedical Engineering Department, University of Connecticut, Storrs, CT 06269, United States of America; 4 Institute of Materials Science, University of Connecticut, Storrs, CT 06269, United States of America

**Keywords:** spinal cord injury, bladder dysfunction, silicon-based microelectrode array, microstimulation

## Abstract

**Objective.:**

Bladder dysfunction is a significant and largely unaddressed problem for people living with spinal cord injury (SCI). Intermittent catheterization does not provide volitional control of micturition and has numerous side effects. Targeted electrical microstimulation of the spinal cord has been previously explored for restoring such volitional control in the animal model of experimental SCI. Here, we continue the development of the intraspinal microstimulation array technology to evaluate its ability to provide more focused and reliable bladder control in the feline animal model.

**Approach.:**

For the first time, a mechanically robust intraspinal multisite silicon array was built using novel microfabrication processes to provide custom-designed tip geometry and 3D electrode distribution. Long-term implantation was performed in eight spinally intact animals for a period up to 6 months, targeting the dorsal gray commissure area in the S2 sacral cord that is known to be involved in the coordination between the bladder detrusor and the external urethral sphincter.

**Main results.:**

About one third of the electrode sites in the that area produced micturition-related responses. The effectiveness of stimulation was further evaluated in one of eight animals after spinal cord transection (SCT). We observed increased bladder responsiveness to stimulation starting at 1 month post-transection, possibly due to supraspinal disinhibition of the spinal circuitry and/or hypertrophy and hyperexcitability of the spinal bladder afferents.

**Significance.:**

3D intraspinal microstimulation arrays can be chronically implanted and provide a beneficial effect on the bladder voiding in the intact spinal cord and after SCT. However, further studies are required to assess longer-term reliability and safety of the developed intraspinal microstimulation array prior to eventual human translation.

## 1. Introduction

Approximately 300 000 individuals are currently living with spinal cord injury (SCI) in the United States with annual incidence of 54 cases per one million, or about 17,810 new cases annually [1]. Restoration of bladder and bowel dysfunctions has the greatest priority for paraplegic people living with SCI, and it is higher than regaining walking ability [2]. Intermittent catheterization is presently used for emptying the paralyzed bladder, but has numerous side effects, most notably, the development of urinary tract infections and chronic cystitis [3–5]. Furthermore, intermittent catheterization does not address the underlying loss of volitional control of micturition. The approach based on electrical microstimulation inside the spinal cord may provide such volitional control. In our earlier study using the cat model of SCI, targeted electrical microstimulation in the dorsal gray commissure (DGC) was shown to be effective in triggering physiologically coordinated bladder contraction and relaxation of the external urethral sphincter (EUS) [6]. Microstimulation-induced voiding was elicited in the majority of the animals (15 out of 22) for a period up to 14 months although the voiding efficacy was rather low in most animals. Twenty animals in that study were implanted with a microwire array with 2D electrode distribution, while only two had relatively thin silicon probes in the array with 3D electrode distribution. We hypothesized that 3D electrode distribution would provide better access to the spinal gray matter nuclei, particularly in the DGC area and, therefore, would facilitate better targeting with lower current levels and reduced current spread to unrelated nearby neuronal groups [7, 8]. Other studies have attempted to achieve neuroprosthetic bladder control with individual spinal microwires [9], spinal microwire arrays [10], spinal epidural stimulation [11–15], and stimulation of the spinal, pudendal, and pelvic nerves [16–19]. Intraspinal microstimulation following SCI has also been used for restoring limb function, including microwires [20–27], microwire arrays [28–31], and multisite silicon arrays [32, 33]. Comprehensive reviews have been published describing various neuroprosthetic approaches for restoring the bladder control after SCI [27, 34–36] and for restoring limb function after SCI [8, 10, 37, 38].

The goal in this study was to develop the next generation of chronically reliable multisite stimulating probes, to optimize the methods of assembling these probes into 3D arrays, and to evaluate the functionality of the array. For probe fabrication, we chose to use the deep reactive ion etching (DRIE) process, which allows a probe thickness of 75 *μ*m (or more), and custom-designed tip geometry for more reliable insertion through thick pial membrane that covers the dorsal surface of the spinal cord. This is in contrast to the probe thickness of 15 *μ*m fabricated using wet etching in our previous study [6], which likely was too fragile for chronic implantation in primates without using a cumbersome mounting device for insertion [39], or to increasing the probe thickness to 80 *μ*m without custom-shaping the tips [40]. The 3D microfabrication technology is also potentially superior to microwire fabrication by facilitating computer-aided design of the probe and tip geometry, better reproducibility, scalability, on-the-probe integration of electronics, and reducing the tissue microtrauma caused by an array insertion due to a smaller number of shanks in multi-site probes as compared to single-site microwires with a similar density of electrodes in the DGC area [6]. Optimization of the array design was done by assessing possible failure modes in the array prototypes during their semi-chronic implantation (up to 6 months). At the end of the study, we achieved an improved array design, and further studies would be required to assess its longer-term reliability over 1 year or longer implantation duration.

## 2. Methods

### 2.1. Probe fabrication

**Figure 1.**
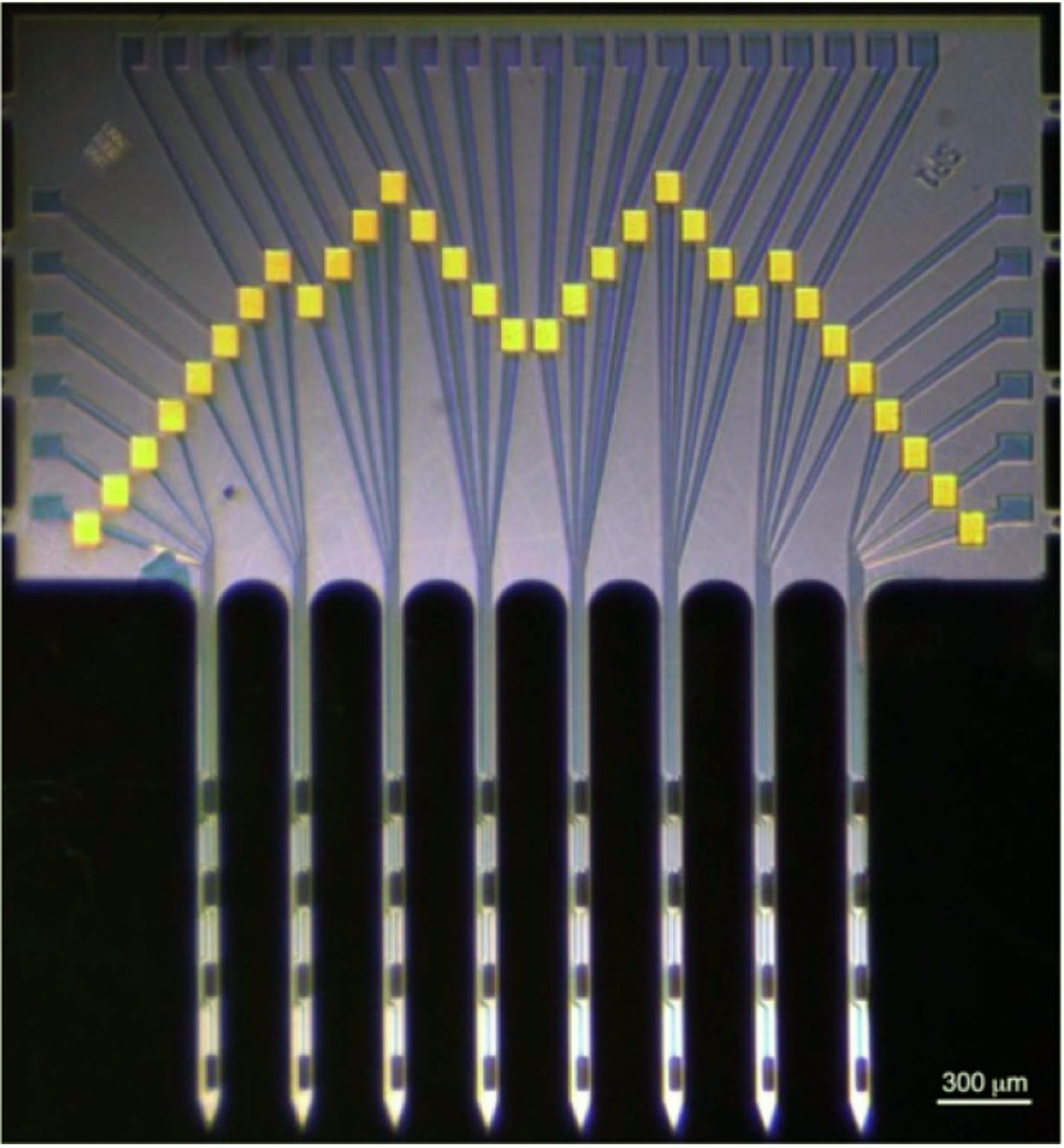
Photograph of a probe with a 4 × 8 microelectrode configuration (lower half). Yellow pads are wire-bonding sites.

The probe consisted of eight 50 *μ*m wide shanks spaced 300 *μ*m apart (center-to-center), with each shank containing four 20 × 100 *μ*m stimulating sites, for a geometrical area of 2000 *μ*m^2^, spaced 300 *μ*m apart (center-to-center) for equal site distribution in the transverse spinal cord plane dimensions (figure 1). The bonding pads in the probe superstructure were wedge-bonded with exposed ends of Parylene-insulated 90% platinum/10% iridium wires in the subcutaneous cable (Model 747677E, West Bond Inc. Anaheim, CA). These Pt/Ir wires were then connected to a long extension cable and to the head-mounted connector (length 70 cm). The cable contained 33 silicone-insulated wires isolated with the outer diameter (OD) of 0.8 mm, or 20 perfluoroalkoxy-isolated wires with OD of 0.2 mm (Cooner Wire, Chatsworth, CA) in a later version of device, with the wires being helically wound using a custom-made winding machine. Of the wires, one was connected to a platinum ground plate.

The probes were fabricated at the Micromachining laboratory at the California Institute of Technology according to the steps previously described [41, 42]. Four-inch diameter silicon-on-insulator wafers were used, and the main insulation layers were silicon dioxide–silicon nitride–silicon dioxide. The tips of the probes were mechanically ground in order to reduce tissue dimpling and facilitate insertion into the spinal cord [41]. Iridium oxide (IrO_x_) was electroplated onto the gold electrode sites to form electrodeposited IrO_x_ film (EIROF), which increased the sites’ charge injection capacity and resistance to corrosion and dissolution during stimulation, compared to pure iridium and platinum [43]. A positive output bias of 400 mV (with respect to the ground electrode) was applied to each of 32 stimulating electrode sites in order to maximize the charge capacity of EIROF during pulsing *in vivo* [44]. Controlled current pulsing for determining charge injection density was done using charge-balanced, biphasic pulses at the intensity of 50 *μ*A. This produced a total potential excursion of *≤±*0.9 V versus Ag/AgCl. The stimulus was administered using a custom stimulator and a control program written in QuickBasic.

### 2.2. Array assembly

**Figure 2.**
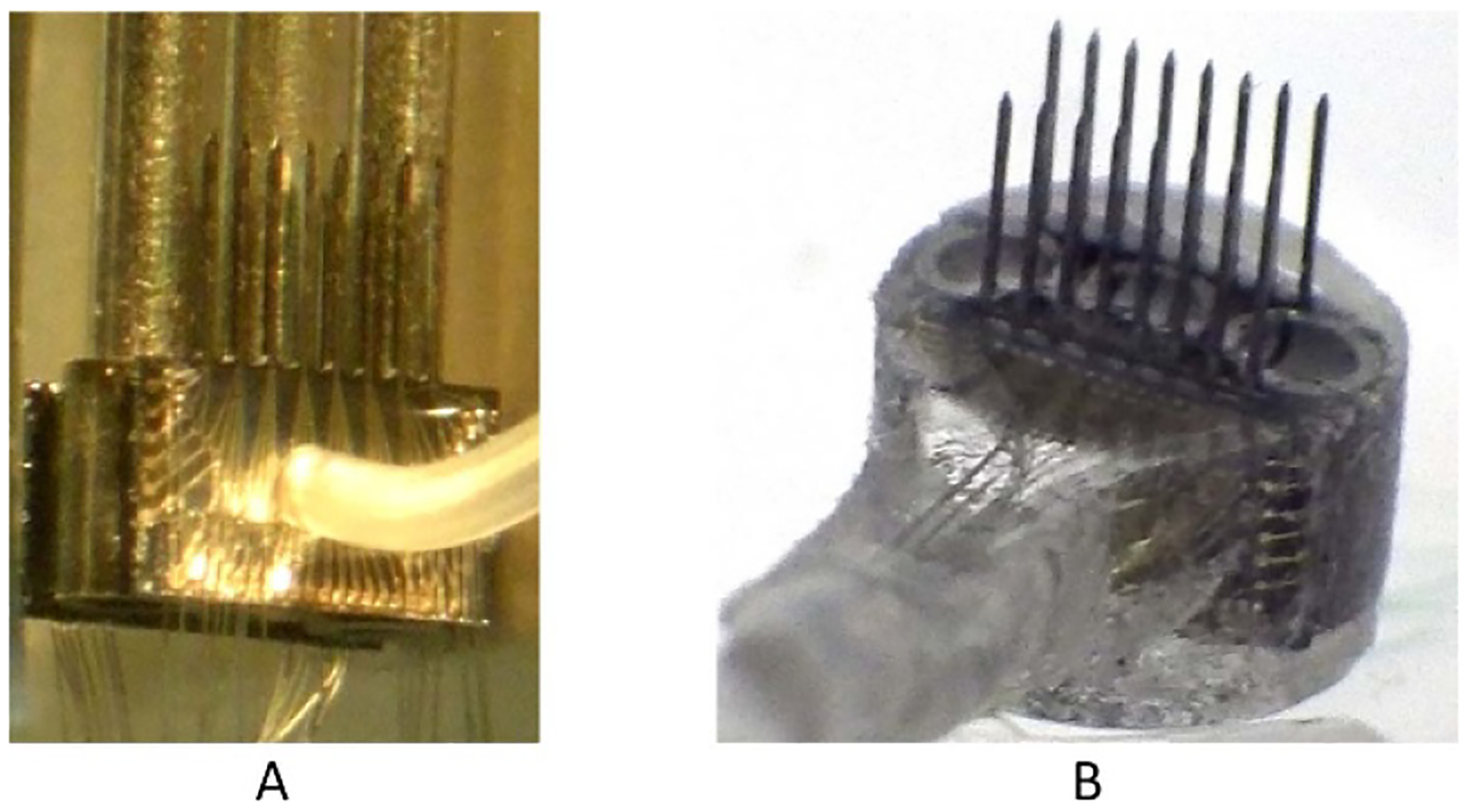
(A) Photograph of a 64-channel array assembly with two planar probes and a three-rod holder for suspending three spacers between the probes; (B) photograph of the released array.

Two probes were assembled into an array, forming a total of up to 64 channels (figure 2). The array’s superstructure was protected from the saline environment by silicone elastomer and epoxy. Similar double-layer polymer coating was used for protecting our earlier microwire and silicon-based arrays, and demonstrated excellent long-term viability *in vivo* [6]. The assembly process was developed, as shown in figure 2. It included three tubular spacers between the two probes and three long rods for precise alignment of the probes (figure 2(A)). Polyvinyl alcohol was used to temporarily hold the parts together while the array was cast and cured in the molding chamber filled with epoxy (EpoTek 310, Epoxy Technology). The array with an attached helical cable was then released from the molding chamber (figure 2(B)).

### 2.3. Electrical testing

Prior to the implantation and at monthly intervals after implantation, the electrical testing of the array was done to confirm the array and cable functionality. The testing was done by delivering biphasic 10 *μ*A current pulses and measuring the initial resistive component (the access resistance) of the access impedance response for each electrode site to provide information about the electrode–tissue interface and the condition of the electrode site, as previously described [45, 46]. Any access resistance values >200 kΩ indicated an electrode failure (open connection or reduced electrode surface). For automating the access resistance testing, a 32-channel Reed relay switchbox (Model SeaI/O-440N, SeaLevel, Liberty, SC) was used for switching among 32 electrode sites; it was controlled by a custom Visual Basic program via the USB connection.

### 2.4. Array implantation procedures

Young adult male cats weighing 2.5–4 kg were used. Animal studies were conducted according to the NIH guidelines and were approved by the HMRI Animal Care and Use Committee. Prior to surgery, the array and some surgical and testing tools (custom insertion tool [47] with hex wrenches, cable for perigenital stimulation, ball electrode for recording of evoked potentials, and a connector cable for pressure transducer and a bladder catheter for urodynamics) were sterilized in ethylene oxide for 24 h using a room-temperature system (Anprolene AN74i, Andersen Products, Haw River, NC). Other surgical tools were sterilized by autoclaving. The cats were castrated to help prevent urethral blockage and to facilitate cleaning of the perigenital region. Three days prior to the surgery, blood work (complete blood count and clinical chemistry) was done to ensure that blood parameters, and the kidney function specifically, were within normal limits. Before the surgery, the cats were given a health examination to check basic physiologic parameters and overall well-ness. Food was withheld 12 h prior to the procedure, but water was offered *ad libitum*. In the morning of the surgery, 20 000 U kg^−1^ of Penicillin G Procaine or 4–7 mg kg^−1^ of enrofloxacin (Baytril) were administered i.m. and repeated 6–8 h post-surgery. Trans-dermal Fentanyl patch (25 *μ*g h^−1^) was placed on a shaved area of the over the ventral chest region. A preoperative anesthetic cocktail (1 ml) containing ketamine (100 mg), acepromazine (10 mg) and atropine (0.54 mg) was administered i.m. Maintenance anesthesia was isoflurane (1%–3%) and nitrous oxide (50%) with balance of oxygen administered by inhalation via an endotracheal tube. The animal was placed on a heating pad at 102°F and covered with drapes to maintain body heat. Sterile saline solution was administered i.v. at a rate of 10 ml kg^−1^ h^−1^ for the 1st hour, then at 5 ml kg^−1^ h^−1^ throughout the surgery. Animals’ vital signs were monitored, including the respiration rate, blood pressure, end tidal CO_2_, heart rate (with ECG), body temperature (with rectal probe), and toe pinch reflex response. The animal’s head was positioned in a stereotaxic frame and lidocaine ointment was applied to the ear bars prior to their use to provide local pain relief.

**Figure 3.**
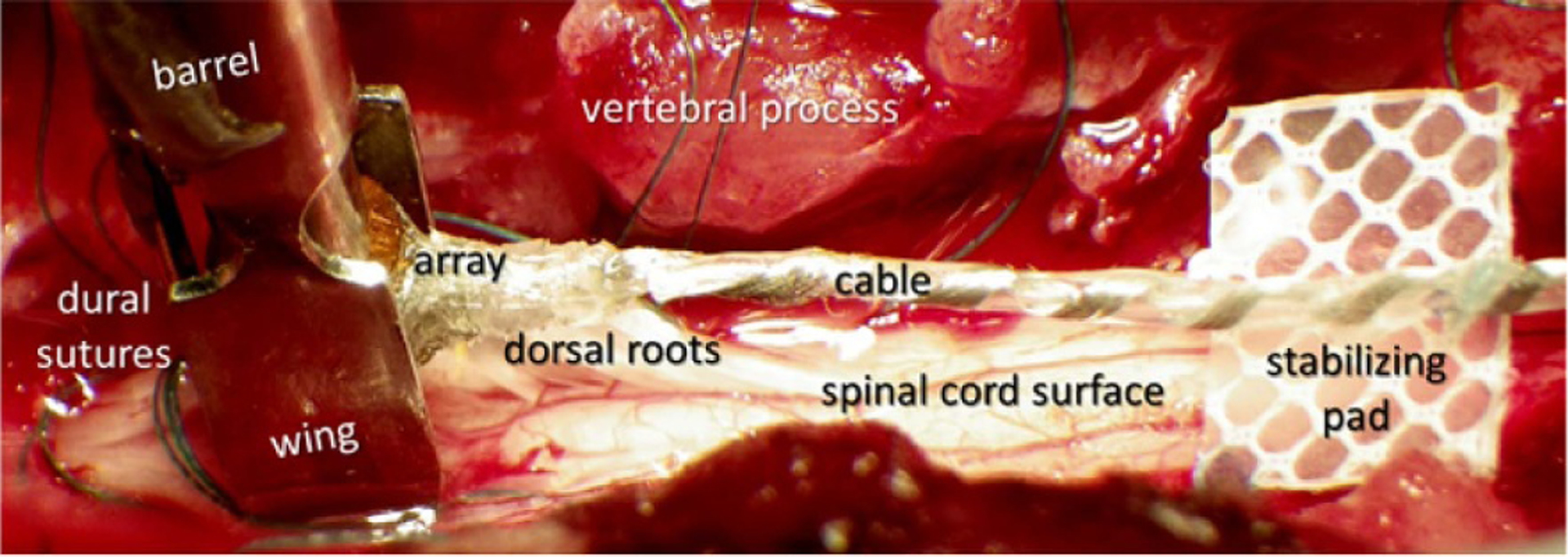
Photograph of the spinal cord laminectomy with the barrel and wings of the inserter tool with a loaded array on the left and the stabilizing pad of the array cable on the right.

A bladder catheter was inserted; a pressure transducer was filled with sterile saline and connected to the bladder catheter. The L4-S1 dorsal vertebral processes were palpated and marked on the skin. Skin and underlying muscle were cut, and the L6 and L7 dorsal spinal processes were exposed and removed, followed by a dorsal laminectomy. Localization of the rostrocaudal spinal cord level involved in the control of micturition (typically S1 or S2 level) was accomplished by intraoperative recording of maximal evoked cord dorsum potential (ECDP) at the spinal cord surface with a ball electrode during electrical stimulation of the perigenital area at 20 mA, 0.2 ms phase^−1^, and 10 Hz using a pair of needles [48]. The evoked potentials were recorded while moving the ball electrode rostrocaudally on the dorsal surface of the sacral cord in 1 mm increments. The DGC location was estimated to be at the maximal ECDP, which was defined as the maximal 2nd peak of the evoked response. The dura over the DGC location was cut longitudinally at midline, the pial sheath was dissected over the dorsal roots, and the roots were gently retracted laterally to expose the spinal cord surface. The ECDP localization procedure was then repeated, while avoiding a con-founding effect of stimulating the dorsal roots. The array was then placed at a bottom of the barrel of our custom insertion tool [47], with the vacuum applied through the barrel to keep the array in place (figure 3). The barrel was positioned at the estimated DGC location using a manipulator mounted on the stereotaxic frame with the side wings barely touching the cord to keep the dorsal roots retracted. The sensing probe is a rod located alongside the barrel of the inserting tool, which was used to detect an initial contact with the dorsal surface of the spinal cord, when lowering the barrel with loaded array. The electrode cable was routed rostrally and attached to the L5 process with a suture to stabilize the cable during the array insertion. Then, a stabilizing pad, made from polyester mesh and attached to the cable 10 mm from the array superstructure, was tamped onto the dura to limit cable migration and reduce the transmission of torque and longitudinal pulling forces from the cable to the array.

The inserter tool was deployed to inject the array into the spinal cord at a velocity of approximately 1 m s^−1^, and, due to its use of the sensing probe, its motion stopped just before the array superstructure impacted the cord surface. Completeness of the array insertion was confirmed using a surgical microscope with built-in video camera (S21, Zeiss) by observing the array superstructure resting directly on the pia. Next, absence of mechanical damage and completeness of insertion was confirmed with the access resistance measurements from the shallowest (most dorsal) electrode site in each shank, as described in section 2.3. Large access resistance values (>200 kΩ) indicated either a shank fracture or incomplete insertion, which was further distinguished by measuring the access resistance of the deeper-located electrode sites. The dura was fully closed over the array with several nonabsorbable 7–0 sutures. The electrode cable was tunneled subcutaneously to the skull using a guiding metal tube. The percutaneous connector was mounted on the skull with four stainless steel screws and bone cement (methyl methacrylate). The muscle and fascia were closed over the spinal laminectomy and around the percutaneous connector on the head using 4–0 monofilament absorbable suture. The skin was closed using 4–0 non-absorbable nylon suture. The animal was removed from the stereotaxic frame, and surgical anesthesia was discontinued. Intraoperative urodynamic testing was then performed while the animal was under light propofol anesthesia, as described in the next section. After the urodynamic testing, the bladder was emptied by draining the catheter to the collection bag. Once breathing on its own, the cat was placed in a heated incubator for recovery from anesthesia, and was monitored until regaining sternal recumbency.

During the first three postoperative days, cats received 0.01 mg kg^−1^ of analgesic Buprenorphine (s.c., every 6 h). Cats were attended daily for general inspection and cleaning the head connectors. Baytril (enrofloxacin, 22.7 mg tablets given orally) or Clavamox (amoxicillin, 62.5 mg tablets given orally) were given in alternation twice a day for 7–14 d after the surgery, as a prophylactic antibiotic. Appetite, behavior, urination, defecation, and any potentially pain-related behavioral signs, such as immobility or reluctance to move, abnormal posturing, decreased appetite, anxiety, aggression, and vocalization were monitored daily. For the first two weeks after surgery, animals were housed in a cage without perches to reduce spinal mobility and allow the array to become stabilized by the growth of connective tissue.

### 2.5. Procedures for spinal cord transection and animal care

The spinal cord transection (SCT) at T_12_ vertebral level was performed in one cat with good urodynamic responses to microstimulation. Pre-surgical procedure was the same as for the array implantation. Spinal cord was exposed by a dorsal laminectomy. A dorsal midline skin incision was made from T_11_ to T_13_, the muscles were retracted, the T_12_ dorsal vertebral process was removed, and a laminectomy was performed. The dura was exposed for a length of about 15 mm. About 0.5 ml of 1% lidocaine was topically applied and injected into the cord. The thin and blunt-tipped 5 mm wide Teflon strip was passed under the cord. The ends of the Teflon strip were lifted slightly and the spinal cord was cut with dura-cutting scissors. The dura was then closed with nonabsorbable 7–0 sutures and covered with a piece of Gelfoam to promote growth of connective tissue. Overlying fascia and skin were closed in layers.

The cat was housed in a fiberglass enclosure with the floor measuring 3′ × 3′ lined with incontinence pads that were replaced several times a day as needed to keep them dry and prevent pressure sores and avoid soiling of the animal. To promote physical activity, the cat was exercised in specially constructed runs made from polyvinyl chloride tubing and polyethylene mesh and lined with foam matting enclosed in vinyl and covered with incontinence pads. Once a week, the run walls were dismantled and cleaned in the cage-washer or by hand steaming. After the SCT, the cat did not clean or groom the parts of its body below the lesion, so the paralyzed hindquarters were daily washed, dried, brushed, exercised, and massaged to avoid joint fixation to substitute for normal grooming. Bladder catheterization or tactile induction of spontaneous bladder and fecal emptying was performed at least twice per day, and urine was collected and tested using urine reagent strips (MultiStix 10 SG, Siemens Medical Solutions, Malvern, PA) for presence of blood and for possible urinary tract infection.

### 2.6. Urodynamic assessment during microstimulation

In spinally intact animals, assessment of urodynamic responses to intraspinal microstimulation was conducted at 1 month intervals, while in one animal with the SCT, the assessment was done at weekly intervals. The cats were anesthetized with propofol, and the urinary bladder was catheterized. A low level of propofol anesthesia was maintained with the animal breathing on its own and responding to strong sensory stimulation. This allowed monitoring of possible undesired effects of stimulation, such as hindlimb flexion, movement of the tail, or pain-ful sensation. Hydrostatic pressure within the bladder vesicle was measured with a pressure transducer (Model 041500503A, Maxxim Medical, Athens, TX). The bladder was filled with sterile warm (37 °C) saline to a bladder pressure of 15–20 mmHg, which is similar to a storage phase of the micturition cycle and is below the threshold to generate reflex contractions of the urinary bladder. The constrictive force, or tone, within the EUS was measured with a second pressure transducer as an ‘infusion pressure’, the resistance to the infusion of saline through a port on the side of the catheter at a rate of 100 ml h^−1^. The EUS was localized by slowly moving the catheter along the urethra to the point of maximum infusion pressure. Data from the pressure transducer was amplified, digitized at 100 samples per second using a 12-bit data acquisition system (USB-6259, National Instruments), and displayed and stored on a computer using a custom Visual Basic program with Measurement Studio ActiveX components. Selection of the electrode sites and pulsing parameters was done using a custom Visual Basic program controlling digital and analog output channels of the data acquisition board. These output channels were used to drive the custom-built current-controlled stimulator. Cathodic-first biphasic pulses were applied to one electrode site at a time at a duration of 0.15 ms per phase, an amplitude of 50 *μ*A, and a frequency of 20 Hz. A train of electrical stimuli was delivered for 30 s, followed by a recovery interval of 60 s before the next train. Charge per phase and charge density were maintained within 7.5 nC phase^−1^ and 375 *μ*C cm^−2^, respectively, in order to prevent stimulation-induced neural injury in the feline sacral spinal cord [48]. Changes in the bladder pressure and EUS tone were calculated as the differences in the average pressures measured during 30 s of stimulation and during 30 s immediately before the stimulation (the baseline period).

After measuring the EUS tone and bladder pressure response to microstimulation, the bladder was filled with saline warmed to 37 °C to a pressure of about 30 mmHg, and the catheter was removed from the urethra to evaluate efficacy of the most effective electrode sites in inducing micturition. Voided fluid was collected into a plastic dish and its amount was measured with top-loading balances (Model PB-S, Mettler-Toledo, Columbus, OH). The voiding duration and voided amount were recorded. A new urethral catheter was re-inserted, through which residual urine in the bladder was aspirated with a syringe. The recorded volume was used to evaluate the voiding efficacy, calculated as a ratio of the voided urine to the total bladder volume (voided plus residual volumes). ‘Near-complete voiding’ was defined the voiding efficacy *≥*67%. The cat with SCT was tested for voiding efficacy without anesthesia, since it did not have any sensations associated with the catheter insertion and bladder filling.

### 2.7. Histology and image analysis of the spinal cord sections

At the end of the study, the animals were deeply anesthetized with pentobarbital (Nembutal, 50 mg kg^−1^, i.v.) heparinized (5000 units, i.v.), and sacrificed by transcardial perfusion through the aorta with a pre-wash solution (0.05% procaine hydrochloric acid in PBS) for 30 s, followed by fixative (freshly prepared 4% paraformaldehyde in PBS) for 2 min. In five of the animals, the necropsy was performed to extract the spinal cord segments with implanted array for the histology, and the array position relative to the spinal cord level was validated by a complete dissection of the sacral spinal roots. The array was then carefully removed, and the spinal cord tissue was post-fixed overnight in 4% paraformaldehyde, dehydrated, and embedded in paraffin using an automatic tissue processing and embedding system (Autotechnicon Mono, SEAL Analytical, Mequon, WI). Transverse sections of the paraffin-embedded spinal cord tissue blocks were cut at a thickness of 6–7 *μ*m using semi-automatic motorized microtome (HM 355, Thermo Fisher Scientific, Kalamazoo, MI). The sections were stained Cresyl Violet (Nissl stain) for defining the gray matter boundaries. Some sections were also immunostained with the antibody to neuron-specific nuclear protein NeuN (MAB377, 1:2 K, Chemicon, Temecula, CA) and visualized using chromogen Vector Red (Vector Laboratories, Burlingame, CA). The stained sections were microphoto-graphed using a digital microscope camera (Spot RT, Diagnostic Instruments Inc. Sterling Heights, MI) mounted on a fluorescent microscope (BX41, Olympus, Cypress, CA).

The transverse area of the spinal cord gray matter and spinal cord boundaries were averaged from multiple tracings of the spinal cord sections in these animals, as described in detail previously [6]. Using a custom image analysis program written in Visual Basic, we then determined the tip locations of the four shanks closest to the midline of the spinal cord. Distance from the central canal is scaled in mediolateral dimension based on lateral gray matter boundary to account for spinal cord compression by the array superstructure.

## 3. Results

### 3.1. Probe fabrication and array assembly

Based on our previous mapping study in the feline spinal cord [6], the dorsoventral span of the DGC was determined to be from 0.7 mm to 1.6 mm from the dorsal surface (with the central canal located at 1.6 mm) while the lateral extent of the DGC was 1.0 mm from the cord’s midline. Therefore, the array of stimulating electrodes was designed to encompass an area of 0.9 mm dorsoventrally and 2.1 mm bilaterally, with an equal 0.3 mm spacing of electrode sites in both dimensions. In the rostrocaudal dimension, the two probes were spaced 1 mm apart, based on a precision of ~1 mm during localization of the rostrocaudal spinal level involved in the control of micturition by intraoperative recording of maximal ECDP in response to perigenital stimulation [48]. In order to limit tissue damage, the shank width was set to 50 *μ*m, accommodating the electrode site width of 20 *μ*m and up to three 3 *μ*m wide conductive traces spaced 2 *μ*m apart.

### 3.2. *in vivo* implantation

Eight cats were semi-chronically implanted with the intraspinal arrays for at least 4 weeks, with four cats implanted for 14+ weeks, while their micturition-related responses to electrical stimulation were evaluated (table 1). The probe thickness was increased to 75 *μ*m compared to 15 *μ*m thickness of the NeuroNexus probes implanted in our previous study [6]. There were no shank fractures during the high-speed insertion. In the first two animals, each cable had 33 silicone-insulated wires with the OD of 0.8 mm (in the second animal we also added an outer silicone jacket), resulting in a rather bulky cable with the OD of 10 mm. These bulky cables caused repeated infections and skin erosions, associated with accumulation of the sero-sanquinous fluid along the cable. These infections and erosions were treated by flushing and draining the cable tract with saline and antibiotic solution, but ultimately, these animals had to be sacrificed after 1–2 months post implantation. In subsequent six animals, we used cables with only 20 thin perfluoroalkoxy-insulated wires with the OD of 0.2 mm and a thin (0.2 mm) outer silicone jacket, resulting in a slimmer cable with the OD of 1.7 mm. Since up to 16 wires were used for per probe, only the electrode sites on the central four shanks were wired.

**Figure 4.**
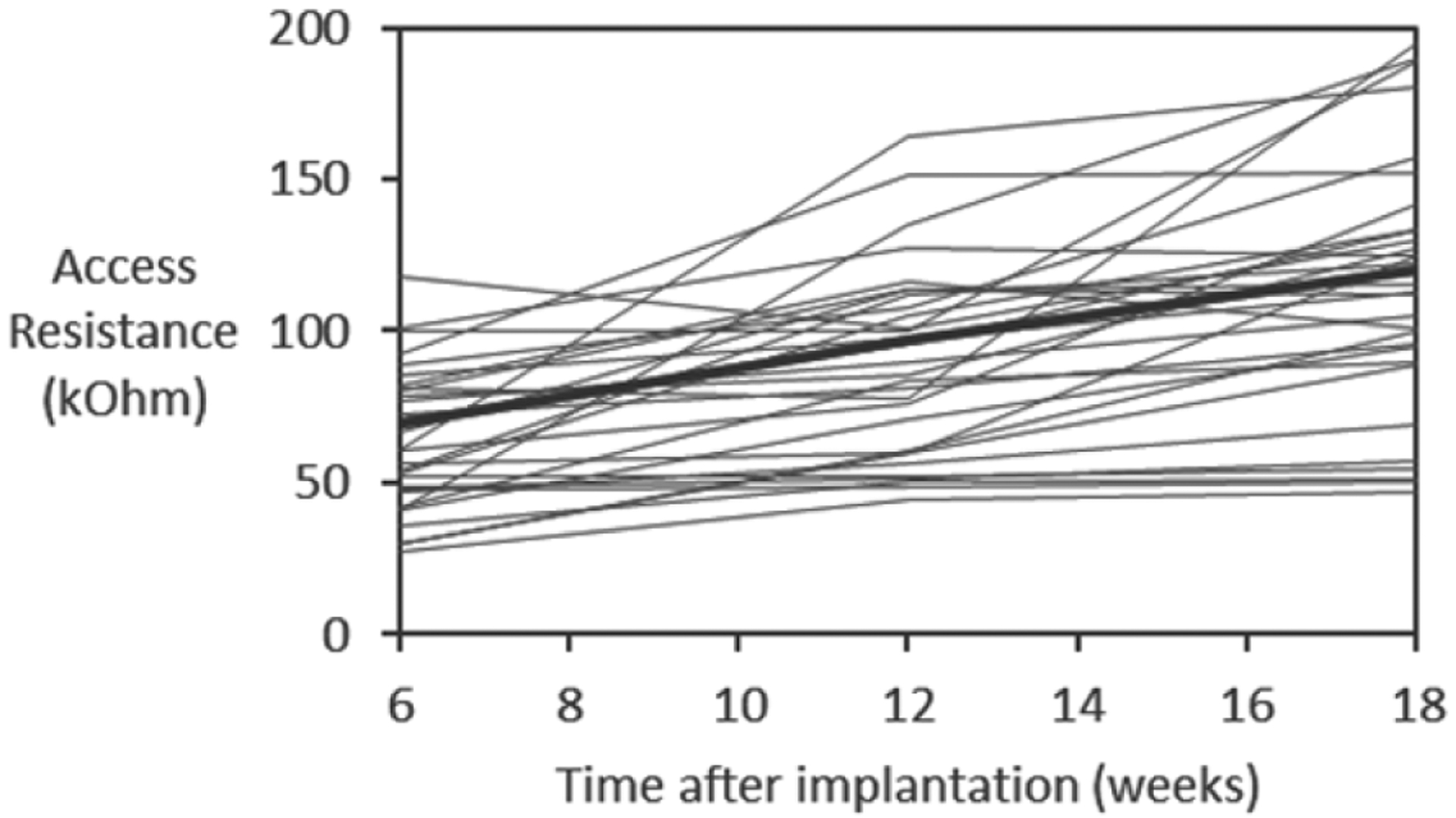
Access resistance of 44 functional electrode sites (<200 kΩ) over the course of implantation in three animals (SP05, SP07, and SP09). The thick line indicates the average values.

These cables and arrays remained electrically functional during the entire period of implantation, as confirmed by periodic measurements of the access resistance of the electrode sites. As evident from figure 4, there was a general trend toward gradual increase in the access resistance over time for the functional electrode sites, likely due to changes in fibrotic encapsulation around the probe shanks. The access resistance remained below a failure threshold of 200 kΩ.

**Figure 5.**
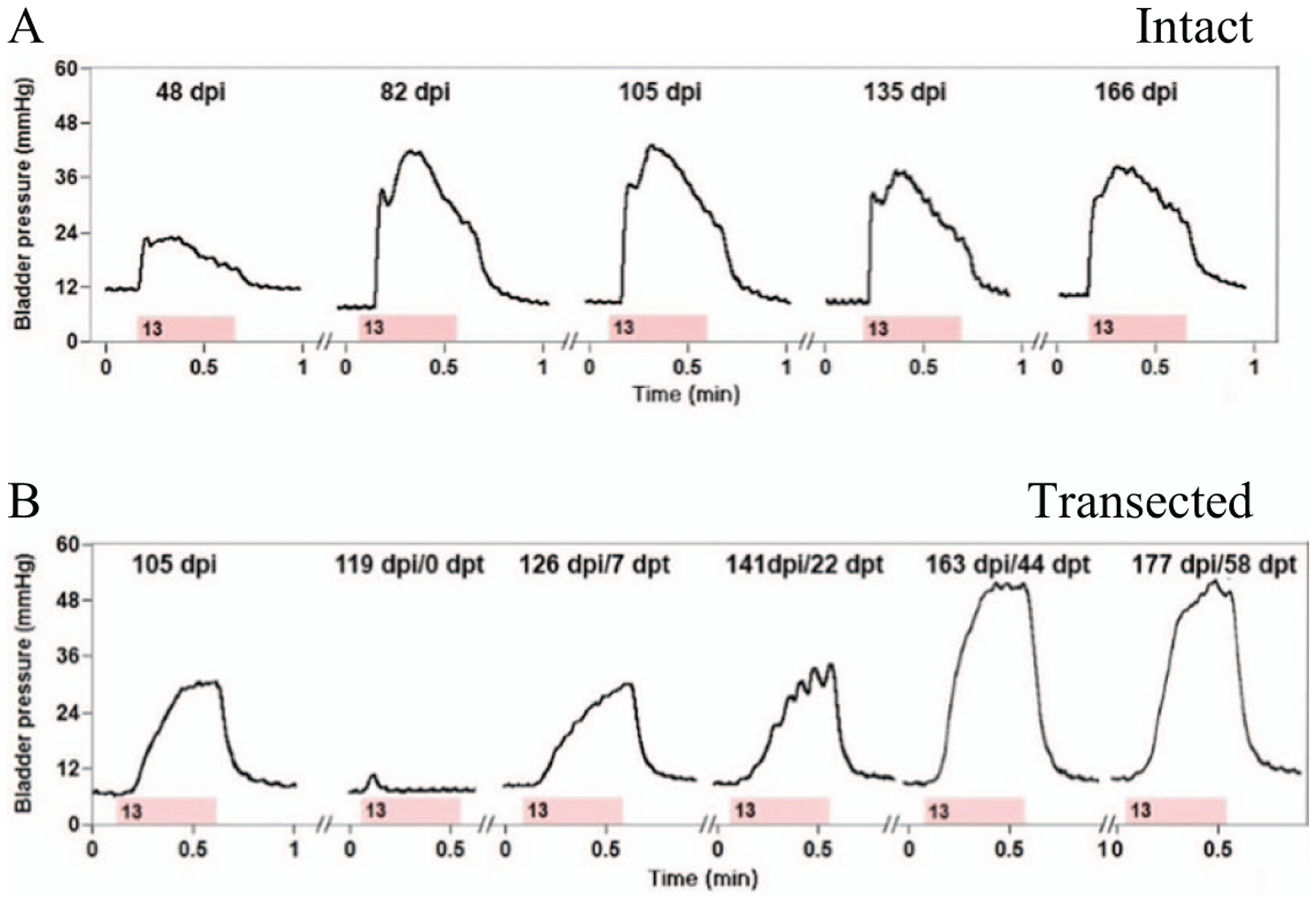
Urodynamic measurements of bladder responses to microstimulation illustrating: (A) stability over 166 d post-implantation, and (B) recovery of bladder control over 58 d after low-thoracic SCT. Abbreviations: dpi—day post-implantation; dpt—day post-transection. The pink bar indicates the duration of stimulation and the number inside the bar indicates the stimulated electrode site.

The effect of microstimulation on the bladder and EUS functions was evaluated urodynamically at an approximately 1 month interval. In all animals, at least one electrode site was effective in inducing bladder responses of at least 10 mmHg. In 4 out of 8 animals, near-complete voiding was induced by stimulation of 1 or more electrode sites. As shown in figure 5(A), the bladder responses to the electrical stimulation sometimes remained stable for a period of 5.5 months post-implantation, however more typically they gradually reduced starting at 6–8 weeks post-implantation.

**Figure 6.**
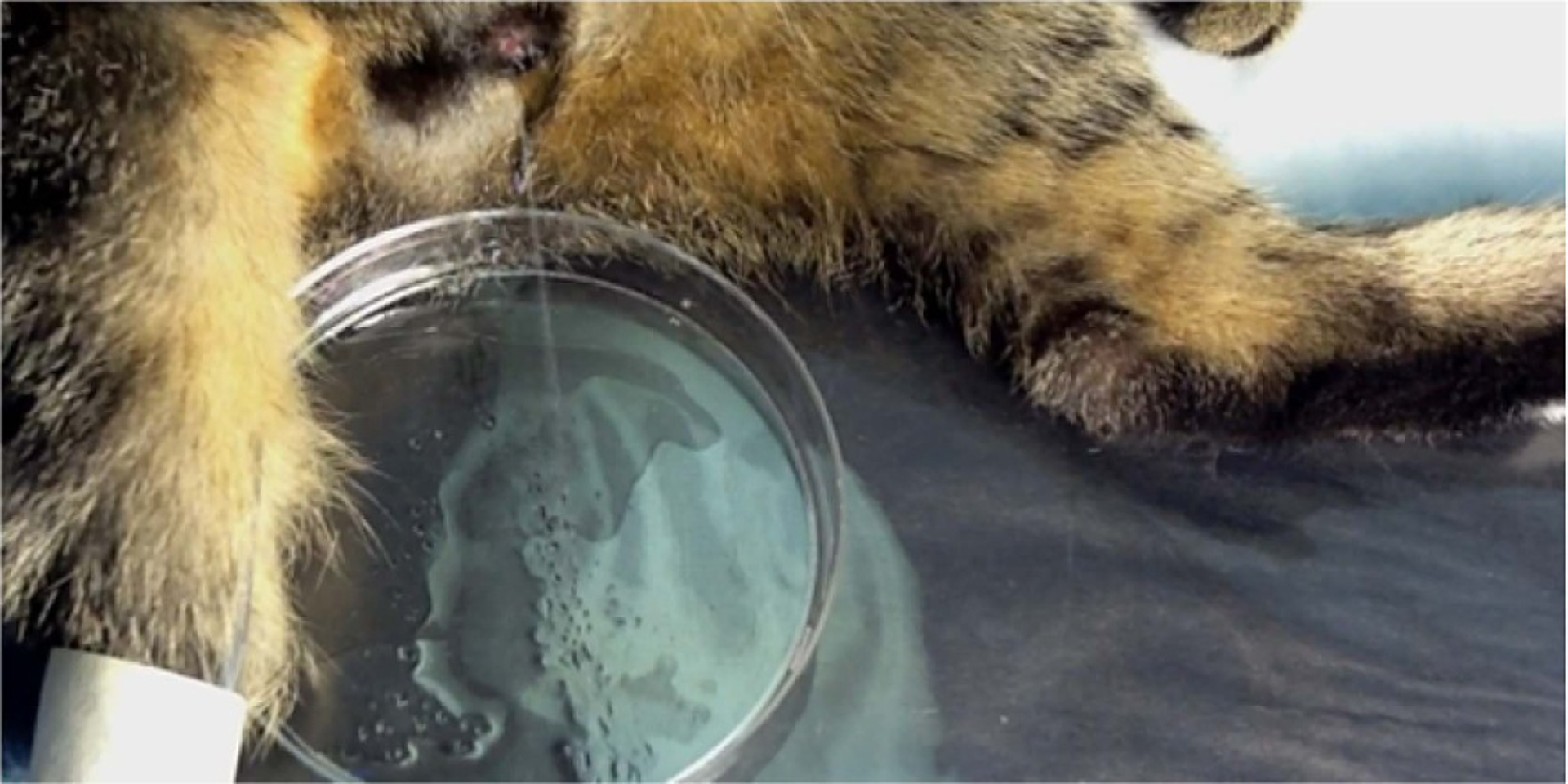
Photograph of bladder voiding induced by stimulating a single electrode site.

A low-thoracic SCT was performed to evaluate the effects of microstimulation in the absence of supraspinal control. Only one animal (SP05) was selected for SCT after confirming that both the bladder responses and near-complete voiding remained stable for 17 weeks after the intraspinal array implantation (figure 5(B)). It was important to establish the stability prior to performing SCT given a progressive loss of electrical stimulation effectiveness over time in most animals (table 1). There was an initial period of acute areflexia (days 1–5 post-SCT), requiring daily manual expressions of the bladder. By day 7 post-SCT, the animal recovered spontaneous bladder contractions and was able to respond to microstimulation. By day 22 post-SCT, the animal developed hyperreflexive non-voiding bladder contractions. At days 44 and 58 post-SCT, the stimulation-induced contractions became stronger than pre-SCT. At day 58 post-SCT, we also examined the effect of the stimulation on the bladder voiding by filling the bladder with saline and then removing the catheter to allow unobstructed flow of urine through the urethra. As seen in figure 6, a stream of urine flow has been induced by stimulation of a single electrode site that was most effective in activation of the bladder and relaxation of the EUS. During the 2.5 min of electrical stimulation, the volume of voided urine was 77 ml, while the volume of residual urine was 27 ml, indicating the voiding efficacy of 74%. In three other animals with near-complete voiding in early weeks post-implantation (SP06, SP07, and SP09—see table 1), efficacy of stimulation reduced over time and was completely gone by 10 weeks post-implantation, so they were sacrificed without performing SCT.

**Figure 7.**
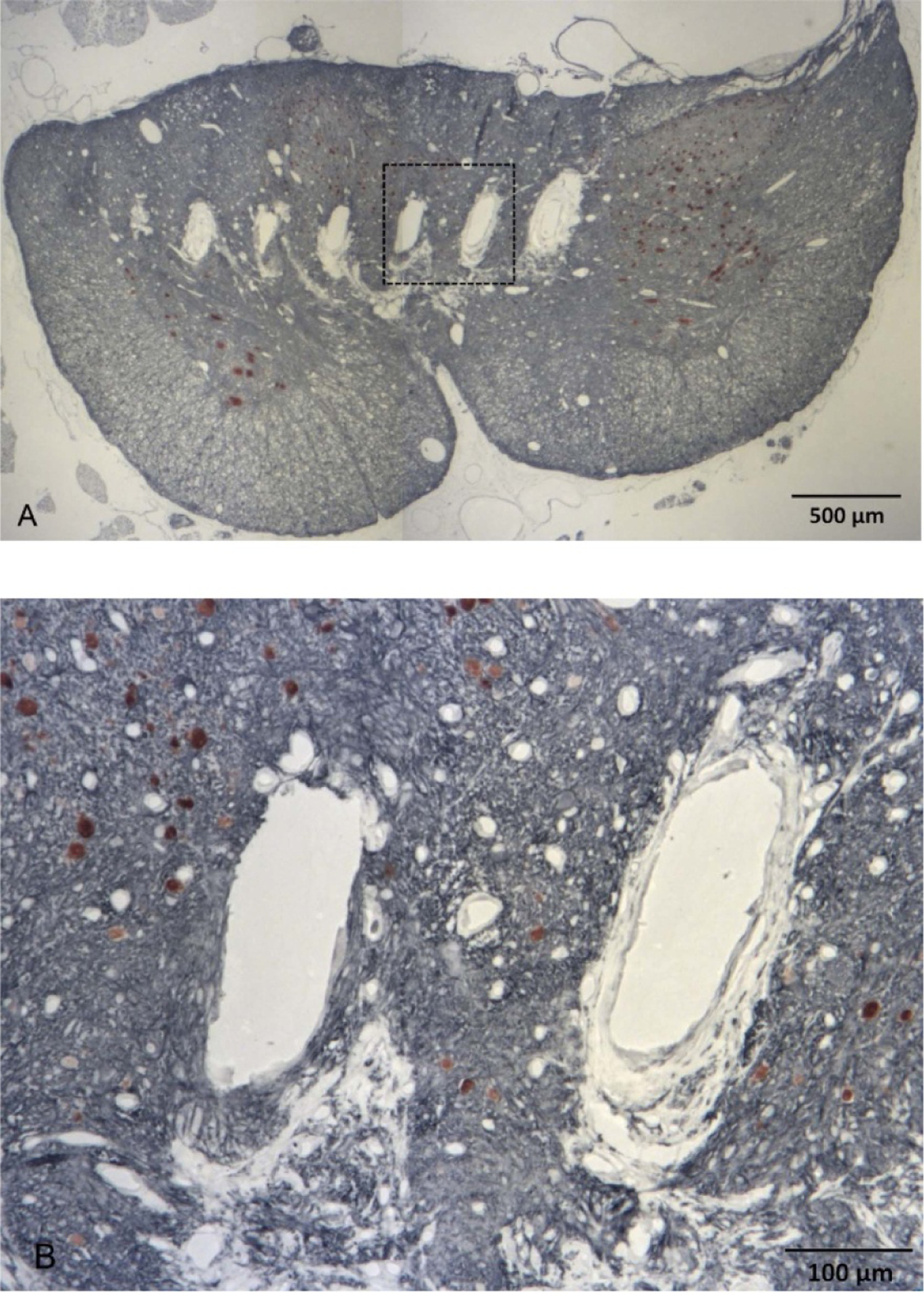
(A) Photomicrograph of eight shank tips in the spinal cord immunostained with NeuN and counterstained with Cresyl Violet. (B) Closeup of two shank tips in the area marked by a rectangle in A.

**Figure 8.**
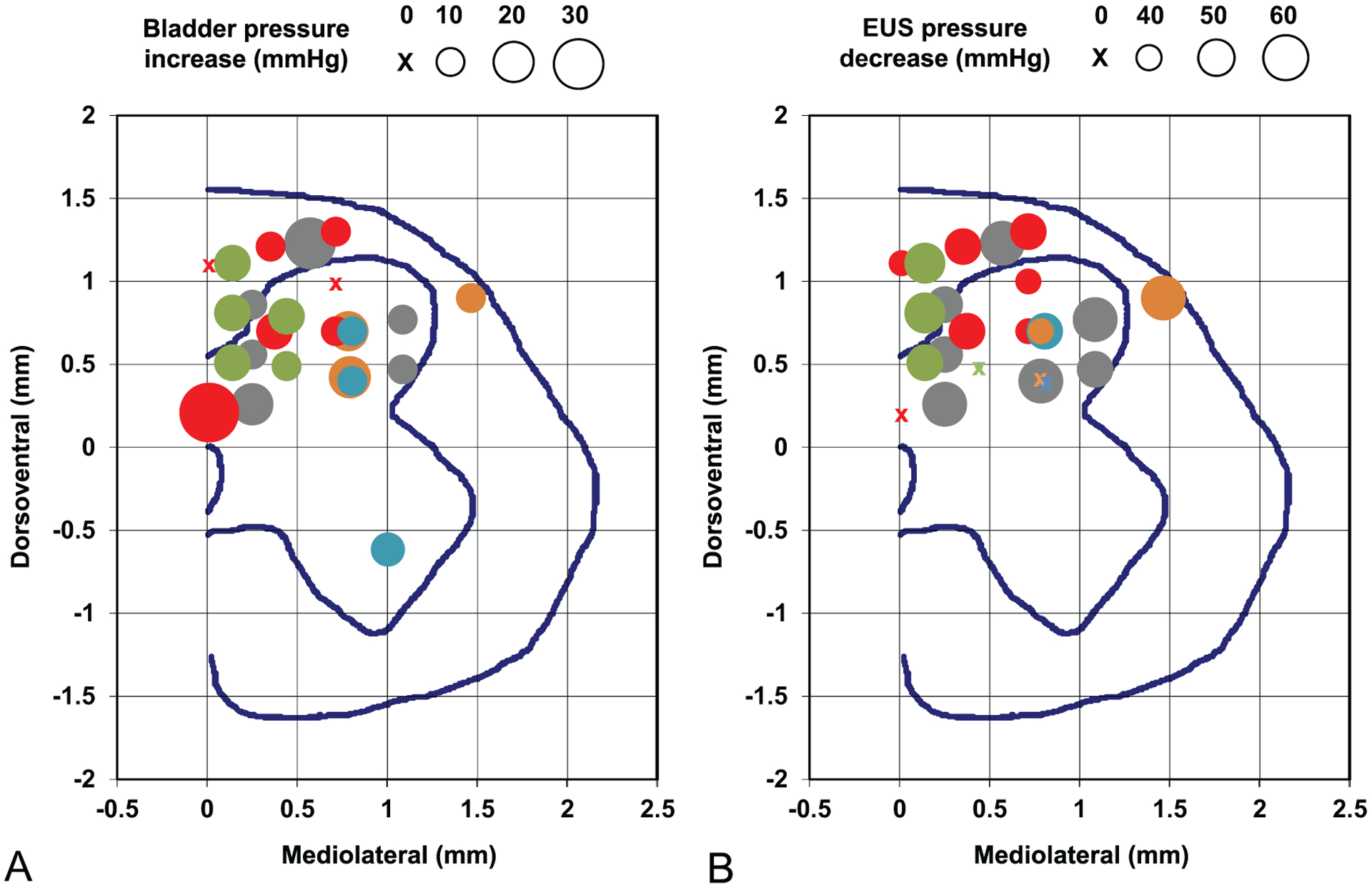
Locations of the electrode sites in the rostral S_2_ spinal segment that produced micturition-related responses: (A) increases in the bladder pressure and (B) decreases in the EUS pressure. The circle size indicates the amount of bladder pressure and EUS pressure change from the baseline level. The circle size scales are shown above the panels. Different circle colors indicate five individual animals (SP05—gray, SP06—red, SP09—green, SP10—blue, SP12—orange). The mediolateral and dorsoventral coordinates are provided in reference to the dorsal edge of the central canal. The blue lines in each panel represent the gray matter and the spinal cord boundaries and the location of the central canal.

After the animals were sacrificed, the necropsy and histology of the spinal cord was performed in six animals that were implanted for 4–6 months (thus discarding two initial animals with bulky cables that were sacrificed after about one month of implantation). Based on the dissection of the sacral spinal roots, we confirmed that the arrays in these animals were implanted at the rostral S_2_ segment of the spinal cord. In five of these animals, spinal cord tissue sections were cut in transverse plane (parallel to the probe shanks); while in one animal (SP07), they were cut in horizontal plane (perpendicular to the probe shanks). As shown on the immunostained spinal cord section in the transverse plane (figure 7(B)), thickness of fibrotic scar tissue around and below the DRIE silicon shafts was rather minimal (25–50 *μ*m) and was similar to that of the scar tissue around NeuroNexus probes used in our previous study [6], despite a greater thickness of the DRIE shanks. The dorsal surface of the white and gray matter was considerably distorted by an indentation produced by a partial sub-sidence of the 1.8 mm high probe superstructure, with a typical displacement of the spinal cord tissue being about 0.5–1 mm (figure 7(A)). Image analysis was then performed to identify the locations of the stimulation sites on the four shanks closest to the midline of the spinal cord (figure 7(A)). These near-midline shanks were selected due to: (a) desire to avoid the lateral gray matter margin containing the motoneuronal nuclei (e.g. Onuf’s nucleus), (b) availability of stimulation response data, as only the central 4 shanks were wired in 6 animals implanted for 4–6 months, and (c) for consistency in comparing with our previous mapping study that primarily utilized the single-site microwires [6]. In these 4 shanks from 5 animals (data from SP07 could not be used due to horizontal plane of spinal cord tissue sections), we plotted 26 (33%) of the electrode sites that induced micturition-related responses: elevation of bladder pressure and/or suppression of EUS pressure (figure 8). The effective electrode sites were located in both the gray matter (DGC area and dorsal horn) and adjacent white matter (dorsal column) (figure 8). The effective and non-effective sites were co-localized, with no discernable sub-location exhibiting higher density of effective sites.

## 4. Discussion

This study confirmed and extended the findings of our previous spinal microstimulation study [6]. It was confirmed that the combination of DRIE process and 3D array assembly technique was suitable for chronically reliable spinal stimulation and for a fracture-free insertion of the arrays through the spinal pia in a moderately large animal (domestic cat), unlike the thinner silicon probes that require special mounting rigs during insertion [39]. It was further demonstrated that the DGC area was involved in the coordination between the bladder and the EUS, as 33% of DGC-located electrode sites in the silicon-based arrays were effective in producing micturition-related responses, which is similar to 29% of effective sites observed with the single-site microwires [6]. In this study, similar to our previous study [6], there was nearly identical effectiveness for the electrode sites in the dorsal horn of the gray matter and in the dorsal column of the white matter. This is not too surprising, considering: (a) considerable current spread from the stimulation sties and (b) similar stimulation thresholds for activating spinal neurons by stimulating in the gray matter and the white matter (refer to figure 5 in [49]). In accordance with our previous study [6], we also observed an increased effectiveness of stimulation in the DGC area for a period of 2 months after SCT, likely due to supraspinal disinhibition of the spinal central pattern generator circuitry for various functions [50, 51] and/or hypertrophy and hyperexcitability of the spinal bladder afferents [52–54]. In addition to taking an advantage of post-SCT amplification effects, the electrical stimulation in the DGC can provide a more physiological approach of recruiting the bladder and EUS motoneurons, since the stimulation initially activates long-endurance/low-force type I motor units followed by incremental recruitment of short-endurance/high-force type IIb fibers [55]. It should be noted, however, that our study did not systematically examine the effects of electrical stimulation outside the DGC, so no conclusion can be drawn about comparative effectiveness of the DGC versus other areas in the dorsal half of the spinal cord.

The issues of glial scarring and foreign-body response to the implanted penetrating probes have been investigated mainly in the cerebral cortex. The spinal meninges are quite similar to the cranial meninges, but contain more collagen, which may serve as a mechanical reinforcer [56]. Similar to the cortex, the electrode implantation causes a range of responses in the spinal meningeal layer, including fibrous tissue thickening [57], elastic softening [58], lymphocyte infiltration [48], foreign-body response with microglial and astrocytic activation [59, 60], and a risk of the cerebrospinal fluid leak [61–64] and hematoma [65]. In this study, the observed chronic foreign-body response was rather minimal, which was possibly due to a subdural placement of the array, stabilizing the array on the spinal surface with the stabilizing pad, and creating a thin uncoiled highly flexible cable segment proximal to the array to promote its free untethered rostrocaudal movement together with the spinal cord inside the vertebral canal.

In this study, we observed considerable indentation of the dorsal spinal tissue following array implantation. Similar spinal indentation was observed after epidural placement of electrode arrays made from shape-memory polymer and Parylene C [66], polyimide and silicone [67, 68], and Parylene-C containing the electronics [69], as well as by penetrating microwire electrodes [24, 70]. The compression force produced by the electrode array (especially, if tethered) may cause the fibrotic scarring and meningeal thickening, creating a physical barrier between the electrodes and the tissue resulting in increased activation threshold and vascular flow disruption. In extreme cases, the compression force can result in the array sinking into the tissue [71, 72]. In the future, the array design would be modified to reduce the height of its superstructure from 1.8 mm to <0.5 mm to reduce the downward force created by elastic connective tissue encapsulation and a limited space inside the vertebral column. The current height was to accommodate mounting of a very large scale integrated chip onto the probe superstructure (unpublished). Follow-up chronic implantation study with a thin superstructure (<0.5 mm) needs to be performed to confirm an absence of noticeable indentation of the dorsal spinal tissue following array implantation in order to consider its potential usefulness as a clinical therapy.

We employed a 3D multisite array with an inter-shank spacing of 0.3 mm and inter-probe spacing of 1 mm. While this is comparable to the shank density of the single-site Utah (Blackrock) array, our device provided an advantage of selecting an optimal depth of the stimulation sites within the complex intraspinal structure. Use of multisite probes with a large number of electrode sites, however, created the challenge of interconnecting to bulky and relatively rigid cables with 33 wires (one cable per each probe) over a 70 cm distance in a large animal, which resulted in repeated infections and skin erosions. That problem can be addressed by designing the probe superstructure for interconnecting it with on-the-probe de-multiplexer chip to reduce the number of wires and/or by reducing the diameter of individual wires (along with their insulation). While the de-multiplexer approach was not tested during semi-chronic implantation, a reduction in the cable bulkiness and rigidity was successful in reducing the infections and skin erosions for the implantation period up to 6 months (unpublished). For longer-term implantations, it would be beneficial to apply the de-multiplexer chip to further improve the cable flexibility.

Overall, over the course of iterative *in vivo* testing of several probe design options, we have arrived at an improved array design and confirmed its functionality during semi-chronic implantation. Further studies would be required to assess its longer-term reliability during 1 year (or longer) implantations in preparation for eventual human translation of the intraspinal microstimulation array technology.

## Figures and Tables

**Table 1.** Description of animals in the semi-chronic implantation study. Abbreviations: BPI—bladder pressure increase, EUSPD—EUS pressure decrease, wpi—weeks post-implantation.

Animal	#cables × wires	Implantation	# (%) BPI > 10 mmHg	# (%) EUSPD > 40 mmHg	Near-complete voiding present	Notes
SP01	2 × 33	4 weeks	0 (0%)	0 (0%)	No	Sacrificed due to repeated infections
SP03	2 × 33	8 weeks	0 (0%)	0 (0%)	No	Sacrificed due to repeated infections
SP05	2 × 20	25 weeks	8 (50%)	8 (50%)	Up to 25 wpi	SCT performed at 17 wpi
SP06	2 × 20	14 weeks	6 (38%)	5 (31%)	Up to 6 wpi	
SP07	2 × 20	23 weeks	6 (38%)	13 (81%)	Up to 4 wpi	Spinal tissue cut in horizontal plane
SP09	2 × 20	18 weeks	6 (38%)	3 (19%)	Up to 10 wpi	
SP10	2 × 20	8 weeks	4 (25%)	2 (13%)	No	
SP12	2 × 20	4 weeks	3 (19%)	2 (13%)	No	
